# Fluid professional boundaries: ethnographic observations of co-located chiropractors, osteopaths and physiotherapists

**DOI:** 10.1186/s12913-024-10738-1

**Published:** 2024-03-15

**Authors:** Joshua Toloui-Wallace, Roma Forbes, Oliver P. Thomson, Nathalia Costa

**Affiliations:** 1 School of Health and Rehabilitation Sciences, Faculty of Health and Behavioural Sciences, The University of Queensland, Brisbane, Australia; 2 University College of Osteopathy, 275 Borough High Street, SE1 1JE London, UK; 3 Faculty of Medicine, Faculty of Health and Behavioural Sciences, University of Queensland’s cLinical Trials cApability Team (ULTRA team), Brisbane, Australia

**Keywords:** Physical therapists, Osteopathic medicine, Chiropractic, Musculoskeletal manipulations, Interprofessional relations

## Abstract

**Background:**

Chiropractors, osteopaths and physiotherapists (COPs) can assess and manage musculoskeletal conditions with similar manual or physical therapy techniques. This overlap in scope of practice raises questions about the boundaries between the three professions. Clinical settings where they are co-located are one of several possible influences on professional boundaries and may provide insight into the nature of these boundaries and how they are managed by clinicians themselves.

**Objectives:**

To understand the nature of professional boundaries between COPs within a co-located clinical environment and describe the ways in which professional boundaries may be reinforced, weakened, or navigated in this environment.

**Methods:**

Drawing from an interpretivist paradigm, we used ethnographic observations to observe interactions between 15 COPs across two clinics. Data were analysed using reflexive thematic analysis principles.

**Results:**

We identified various physical and non-physical ‘boundary objects’ that influenced the nature of the professional boundaries between the COPs that participated in the study. These boundary objects overall seemed to increase the fluidity of the professional boundaries, at times simultaneously reinforcing and weakening them. The boundary objects were categorised into three themes: physical, including the clinic’s floor plan, large and small objects; social, including identities and discourse; and organisational, including appointment durations and fees, remuneration policies and insurance benefits.

**Conclusions:**

Physical, social, organisational related factors made the nature of professional boundaries between COPs in these settings fluid; meaning that they were largely not rigid or fixed but rather flexible, responsive and subject to change. These findings may challenge patients, clinicians and administrators to appreciate that traditional beliefs of distinct boundaries between COPs may not be so in co-located clinical environments. Both clinical practice and future research on professional boundaries between COPs may need to further consider some of these broader factors.

**Supplementary Information:**

The online version contains supplementary material available at 10.1186/s12913-024-10738-1.

## Introduction

Several healthcare professions provide care for individuals with musculoskeletal (MSK) conditions. These include chiropractors, osteopaths and physiotherapists (COPs), who work predominantly in the private sector in Australia (73%, 96% and 97% respectively) [1]. Each profession has its own regulatory board and make up approximately 6% of the registered health workforce in Australia [2]. Consultations with COPs in the Australian healthcare system count in the millions each year [3]. These professions are trained to assess and manage MSK conditions with similar manual or physical therapy techniques including, but not limited to, manual therapy (joint mobilisation, manipulation and massage), exercise therapy, and electrophysical agents [4]. For example, with the exception of medical practitioners, these three professions are exclusively permitted to perform cervical spine manipulations under the National Law [5]. In short, despite their distinct training pathways and professional regulation, there is the potential for COPs working in settings that treat predominantly MSK conditions to have overlapping scopes of practice, meaning that there may be similarities in the clinical approaches, interventions and activities they are licensed to perform [6].

Current overlapping scopes of practice between COPs stem from shared histories and connections to manipulative therapies [7]. While some may anticipate that overlapping scopes of practice would result in commonalities and the altruism on which collaborations might be based [8], others have argued that they hamper collaboration between COPs due territorial behaviour and notions of turf-wars [9]. For example, many health insurance companies combine rebates for COPs together into one pool of funding, leading general practitioners to question the economic viability of COPs replicating similar services [10]. It may be for these reasons, combined with the desire to be seen as relevant and unique, that COPs are rarely co-located within the same MSK clinic. In the few settings where they are co-located, an understanding of the nature of their relationships may shed light on broader professional issues facing COPs.

Our previous qualitative study conducted interviews with COPs who were co-located with each other, and the findings highlighted that some basic collaborative practices may exist [11]. Such practices included cross-referral and joint consultations which were perceived by participants to benefit clients, clinicians, and the clinics they operated within. In this prior study, participants shared that aspects of clinic culture, personal beliefs and attitudes influenced their perceived ability to navigate blurred professional boundaries between themselves and their colleagues in a respectful and productive way [11]. While our previous study provided some evidence of what clinicians’ *report* occurs within their own practice, further investigation into the nature of the professional boundaries in co-located clinics may be more closely explored using different methods such as ethnographic observations [12].

In this current study, we use the term ‘multi-disciplinary co-location’, or simply ‘co-location’, to refer to situations where professionals from different disciplines work within the same facility [13]. While co-location can provide health professionals with additional opportunities for collaboration [13], co-location alone does not automatically guarantee collaboration as there are other structural and behavioural elements that influence the nature and extent of collaboration, such as ease of communication, managing competing priorities or territorialism [14]. Research in some health care settings suggests that intra and inter-professional conflict can negatively affect quality of care [15]. Most co-location literature is, however, in the context of primary care and centred around general medical physicians, and little is known about the nature of co-located MSK professions such as COPs. In such instances where there is an overlapping scope of practice, it may be challenging for COPs to navigate more ambiguous professional boundaries.

Professional boundaries serve as a mechanism for professionals to delineate their specific areas of expertise and define their roles within a clinical environment. These boundaries facilitate the identification of distinct professional categories, thereby aiding in the clear establishment of the scope and limitations of each professional’s role and expertise [16]. We have used the term ‘boundary’ to describe the point at which we perceive the scope or role of one profession to end and another to begin. In this study, we were interested in examining the nature of the professional boundaries between COPs, as well as exploring the ways they carry out what is known as ‘boundary-work’ whilst being co-located. Boundary-work is known as the discursive practices (i.e., anonymous and historical rules that govern knowledge [17]) individuals and groups employ to distinguish themselves from others and assert intellectual or professional authority [18]. Boundary-work has previously been examined in relation to MSK healthcare. For instance, analysis of interviews with practitioners from over 10 orthodox and alternative MSK-related disciplines identified a range of rhetorical strategies practitioners use to distinguish their services from those provided by others [19]. Such strategies included notions of limitation (i.e., ‘other occupations do not possess something we do, hence they are limited’), holism (i.e., ‘we are holistic and others are not’) and prevention (i.e., ‘we prevent problems because we treat the causes, while others only treat symptoms’) [19]. Our research seeks to build on previous research by exploring further examples of boundary-work in the context of clinical practice by focusing on COPs and using ethnography to explore the physical environment and non-human aspects as additional sources of data. Our aim was to understand the nature of professional boundaries between chiropractors, osteopaths, and physiotherapists (COPs) within a co-located clinical environment. We also aimed to explore and describe the ways in which professional boundaries may be reinforced, weakened, or navigated within the social and physical environment of the clinic.

## Methods

### Theoretical stance & methodology

Our research stems from an interpretivist paradigm, with relativist ontological and subjectivist epistemological bases, meaning that we see the nature of the boundaries between COP practitioners are not pre-existing, stable nor waiting to be ‘objectively’ examined and ‘found’. Rather, the subjective realities (i.e. the ontology) of these boundaries are multiple and socially constructed [20]. From this interpretivist/relativist perspective, knowledge and research results are created through the process of interaction between the inquirer and the inquired, i.e., the interaction between the researchers’ subjectivity and participants, within their context (i.e., epistemology) [21]. In alignment with these theoretical bases, we employed ethnographic observations as a methodology to understand the nature of the boundaries between COPs and explore how they are navigated within the clinics where the observations were conducted. Ethnography is concerned with describing people in their cultural context, and how their behaviour, as individuals or groups, is influenced by this cultural context [12]. Contemporary ethnography is not so much concerned with ‘other’ cultures as it is with settings closer to ‘home’ [12]. As members of the professions or ‘cultures’ we researched, our ethnographic approach used participant observation to undertake detailed description, at times questioning that which is taken-for-granted or familiar [12]. This means that we were clear about our role in the research setting and ensured that participants understood this and the purpose of the research. Our detailed descriptions have taken note of the specific temporal, spatial and cultural context within each clinic environment [12].

Both the ethnographic observations and the analysis that followed were informed by two theories of ‘professions’: neo-Weberianism and post-professionalism. A neo-Weberian conception of professions is interested in professional power, their boundaries, and how they compete within the market [22]. From a neo-Weberian perspective, COPs are considered independent ‘social actors’ competing for legitimacy, power, and socioeconomic rewards [23]. Post-professionalism assumes that the future of ‘the professions’ as a social class will be less prominent in the organisation of society [22]. For COPs, this means that not only does post-professionalism challenge the boundaries that separate them, but it challenges the utility of their very existence as independent and powerful social structures, and there have been calls for the professions to be more critically reflexive and responsive to changing societal needs [24]. The primary researcher familiarised himself with these theories prior to going to the field, meaning that he considered elements from these theories during the observations to explore situations where boundaries between COPs were clear or unclear, and how clinicians navigated these boundaries in the clinic.

### Positioning of the researchers

The positioning of the primary researcher (JT) as a male physiotherapist who made the observations is acknowledged in reflexive fieldnotes and during analysis. As a registered physiotherapist observing other physiotherapists, but also chiropractors and osteopaths, the primary researcher is simultaneously an ‘insider’ and ‘outsider’ observing the inter-personal relationships and environment within the clinics [25]. JT acknowledged and considered his positionality during data collection by regularly reminding participants of the aims of the study and reassuring them that he was not there to judge their clinical practice. He also attempted to avoid coming across as ‘intimidating’ during the observations.

The other members of the research team consisted of: a female physiotherapist who has worked clinically in MSK practice in Australia for 17 years and has a research interest in qualitative and health professional education (RF); a female physiotherapist who has worked clinically in both Brazil and Australia for 9 years and has research experience in MSK health and policy, as well as a broad interest in inter-disciplinary practice (NC); and a male osteopath who has worked clinically in the United Kingdom for 16 years and has experience using qualitative research methods to examine professional identities within osteopathy (OT). In alignment with our reflexive approach to thematic analysis, we acknowledge that our collective and subjective experiences were used as resources in the analytical process. Said differently, we negate the idea of an unbiased and objective perspective and therefore, acknowledge that our team attempted to remain critically aware of how our positionality influenced our methods, findings, interpretation, and analysis.

### Participants

Participants were Australian-registered COPs who were co-located in a private clinic with at least one practicing clinician from at least one of the other two professions. Two clinics were chosen based on these requirements. Clinic A was known to the research team due to the clinic’s participation in our previous related study [11] and was purposively chosen based on likely inter-professional interactions within the clinic even though no osteopaths were located there at the time of the study. Clinic B was a multi-disciplinary clinic identified through an online search and direct enquiries and was chosen due to the presence of practitioners from all three professions. The research team did not have a close connection with, or position of responsibility over, any of the participants prior to or during the study. These two clinics were the first and only clinics approached to participate in the study due to their agreeing to participate. If either of the clinics declined, the researchers would have contacted other clinics involved in previous research until the criteria were met across at least two sites. The research team collectively determined that two sites would be adequate to collect sufficient data for the purpose of this study and did not pursue recruitment of other sites.

The primary researcher approached the director of each clinic by email or phone call to invite them and their clinicians to participate. Once they agreed to be involved in the study, the research team sent information to be distributed to all relevant clinicians at the site, outlining what is involved as a participant. The clinic director and all invited clinicians also received a written informed consent form and demographic information form to be completed and returned to the primary researcher at the beginning of the field visit.

The participants of the study were consenting COPs who were working in the clinic during the observations. Occasionally, there were other non-consented individuals in a space that the researcher was observing, such as other clinicians (e.g. podiatrists, massage therapists, etc.), clients in waiting rooms or in a clinical interaction, and administration staff. Anything non-consented clients or staff said or did was not recorded in any detail in the data or subsequent analysis (only relevant general comments that relate to the consenting participants were included in field notes). For these reasons, we did not ask for written consent from clients or other staff who were present during observations of participants, however verbal consent was always obtained for each individual interaction.

### Data collection

The primary researcher (JT), who received training in qualitative research methods, produced research data as field notes through ethnographic observations, including comprehensive notes of interactions and reflections during and at the end of each day of on-site observation. Regular reflexive discussions with the research team (NC, OT, and RF) occurred during the fieldwork to enhance reflexivity and strengthen simultaneous analysis.

The field notes were produced by the primary researcher while physically attending each clinic during working hours (approximately 9am to 4pm) over four consecutive days within one week at each clinic site. The week-long duration of each visit may have improved credibility of the research since the participants may have become increasingly comfortable with the researcher’s presence [12]. The primary researcher attempted to organise the timing of the visits to coincide with increased opportunities for interactions between clinicians, such as in-services between staff, or when multiple clinicians planned to attend to the same client. However, in reality, these were busy clinics and there were no major adjustments made to schedules or plans for the researcher. Therefore, the observations were made during a typical work week.

At the beginning of each observation week, the researcher distributed hard copies of the consent and demographic forms for retrieval by the end of the day, accompanied by introductions and a verbal outline of the study prior to obtaining written consent. While conducting observations, the researcher used the research questions as a framework while still maintaining a level of flexibility based on situations arising at any given time. Generally, the researcher avoided interrupting the clinic’s regular activities as much as possible and was usually quietly positioned in unobtrusive locations, standing or sitting to the side of rooms. Observations were made within several physical spaces, including: the clinic waiting room, hallways, common exercise areas, staff break rooms, and at times within consultation rooms. The researcher took note of various elements in the physical and social environment, for example: what clinicians, other staff and clients say; non-verbal communication; the physical spaces, room, posters and furniture; and the emotional environment, such as tone of voice, body language, facial expressions, tensions and possible misunderstandings. The researcher had opportunities to ask clarifying questions with individual or groups of clinicians, as well as non-clinical staff, as required throughout the day. Occasionally, the researcher would observe joint-consultations where two clinicians work together with a client at the same time, and verbal consent was always gained by clients prior to the researcher entering the space, as per ethical approval.

### Data analysis

In alignment with the study’s theoretical underpinning, our data analysis was informed by interpretivism [21] and was guided by the principles of ‘reflexive thematic analysis’ (RTA) [26]. The key principles of RTA which guided this analysis were: i) acknowledgment of researcher subjectivity; ii) analysis cannot be more or less objective but can be stronger or weaker; and iii) themes do not passively emerge from the data but instead are actively produced by the researchers. For example, throughout the study the researchers questioned their own interest in particular observations and whether these interests would be present if they were aligned with a different professional identity. RTA was chosen as the method of data analysis because it is adaptable to many types of empirical research and is flexible, meaning that it can be done in different ways. For our purposes, we used the following iterative phases:

1. JT wrote both hand-written and electronic observation notes during the fieldwork observations.
2. JT transcribed field notes into a coherent electronic format (see Appendix 1 for observation occasion template).
3. JT discussed the observations and relevant data with the whole research team in regular meetings after each day of data collection and in the months following observations.
4. Drawing from team discussions, JT read and re-read the observations, writing reflective memos on the data while using excerpts and grouping them under evolving themes and taking into account his own positionality throughout the process.
5. Preliminary results were written by JT and refined by members of the research team.

Ethical approval for this study was provided by the (The University of Queensland) institutional Human Research Ethics Committee (2022/HE001568). While designed for interviews and focus groups, the relevant criteria within the COREQ tool was used in the refinement of this manuscript to strengthen qualitative research trustworthiness [27].

## Results

### Sites & participants

The data for this study were generated at two sites: Clinic A and Clinic B. These clinics were located within two large metropolitan cities (with a population of over 1 million each) in two different states of Australia. Due to clinic availability, Clinic A was first visited over one week, followed by Clinic B the following week. For the purposes of anonymity, only general and relevant details of each clinic and participants are described below (see Table 1). No potential participants refused to participate in the study. None of the participants held dual registration for any of the three professions.

Clinic A was established less than ten years ago by three clinicians, a physiotherapist, a chiropractor and a podiatrist. It was located on a suburban main road, within close proximity of other healthcare providers, businesses and restaurants. The physical structure of the clinic was a standalone building with approximately ten closed/private treatment rooms, a small gym area, waiting room, as well as a staff only lunchroom. The clinic is decorated with minimalistic furniture, plants, and art works on the walls. There were 10–15 staff who identified as physiotherapists, chiropractors, podiatrists, massage therapists, as well as an administration team and were not necessarily all present simultaneously. It was a very busy suburban clinic, with clientele predominantly presenting with sporting or work-related injuries and appeared to have a mostly middle-class socio-economic background.

Clinic B was established over 20 years ago by a chiropractor and a medical doctor. It was located on a quiet suburban street among residential homes and a short drive from other healthcare or community facilities. The physical structure of the multi-disciplinary clinic was made up of standalone buildings with separate wings/areas loosely dedicated to different groups of professions: medical doctors (general practitioners) and pathology; physiotherapy and podiatry; chiropractic and osteopathy; occupational therapy and other allied health; remedial massage and administration. Each area had a mixture of private rooms and curtained or open spaces, with many medical or anatomical related posters scattered across most walls, which generally corresponded to the profession which practiced in that area of the clinic. There were over 30 clinical staff from a diverse range of professions and as such the clinic was very busy with predominantly middle-class clientele.

As determined through conversations between the directors of both clinics and the researchers, as well as through observation of clinic procedures and documents, the directors desired strong inter-professional collaboration. In both cases there were policies that attempted to enable this, such as a policy where a patient would only ever pay a single appointment fee (charged at the rate of the main provider) regardless of how many clinicians saw them, or orientation documents for new employees outlining their model of inter-professional collaboration. Observations about the influence of these policies and procedures are considered below.

**Table 1:** Demographic information of participants

Participant number	Profession	Gender	Age Range (yrs)	Clinical Experience (yrs)
1	Physiotherapy*	M	30–39	5–9
2	Physiotherapy	M	30–39	5–9
3	Physiotherapy	M	20–29	0–4
4	Chiropractic*	M	30–39	10–14
5	Chiropractic	F	50+	15–19
6	Chiropractic	M	20–29	0–4
7	Chiropractic	F	40–49	15–19
8	Physiotherapy	M	20–29	5–9
9	Physiotherapy	F	30–39	10–14
10	Physiotherapy	M	20–29	0–4
11	Chiropractic*	F	50+	20+
12	Chiropractic	M	40–59	10–14
13	Chiropractic	F	30–39	5–9
14	Chiropractic	M	50+	20+
15	Osteopathy	M	20–29	0–4
	* Clinic Director	*F = 5/15*		*(Median = 9)*

### Nature of the professional boundaries

Through our observations we interpreted physical, social and organisational boundary objects that influenced the nature of the professional boundaries between the participants. We have adopted the term ‘boundary objects’ as it was coined by Star and Griesemer [28] to describe tangible and non-tangible entities that can be used as a tool to mitigate disparities in language and methodologies when people from different ‘social worlds’ wish to cooperate. While COPs might normally be grouped together within the ‘world’ of professions that use physical therapies to treat MSK conditions, for our study we consider COPs to be from different ‘social worlds’ due to their distinct training and regulatory bodies. Despite the differences in how COPs pursue their work, Star and Griesemer [28] argue that some degree of coherence, common language and standardisation is required to enable these otherwise distinct social worlds to communicate. We also use the term ‘boundary objects’ to denote any physical or non-physical objects or entities that have an influence on professional boundaries.

A key characteristic of the nature of professional boundaries that was interpreted was their fluidity. In almost every instance, a particular physical, social or organisational boundary object had multiple influences on the nature of the professional boundaries. It may have at once reinforced one boundary while simultaneously weakening another– interconnected ‘action/reaction’ type processes. The fluidity of professional boundaries explored in this study is a concept that underpins the illustrative examples articulated below. See Table 2 for a summary of themes, key findings and examples that are explored in the sections below.

**Table 2:** Summary of themes, key findings and examples

Theme	Boundary object and key finding	Examples
**1) Physical boundary objects within the clinic**	**1.1) Clinic floor plan and positioning of rooms** *Arrangement of treatment rooms in the clinic either strengthened or weakened professional boundaries.*	The two clinics had different arrangements that in each case strengthened the boundaries separating COP professions (distinct sections of the clinic for each profession) or weakened the boundaries between COPs (interspersed rooms with doors entering a single hallway).
	**1.2) Large equipment** *Large equipment used by only one profession strengthened professional boundaries.*	Chiropractic ‘drop-tables’ only used by chiropractors, delineating their workspace. General treatment plinths used by the osteopath and physiotherapists. Osteopath further attempted to highlight differences between himself and chiropractors through ideological boundary-work.
	**1.3) Small equipment** *Small equipment stored in certain rooms/areas of the clinics, further reinforced differences between COPs.*	Treatment tools such as activators (chiropractic) and exercise bands (physiotherapy) physically located in respective rooms. Participants also discuss these items in a way to distinguish themselves (their beliefs and practice) from other COPs– performing boundary-work.
**2) Social boundary objects: identities and discourse**	**2.1) Identities** *Dominance of personal identity over professional affiliation of COPs.*	In a dialogue with a chiropractor and a physiotherapist, they both spoke about how they did not focus too much on professional titles. In another example, a physiotherapist reports cross-referring to a chiropractor based on his experience with managing patients with headaches, as opposed to it being solely based on his title.
	**2.2) Discourse** *Words and concepts used between COPs weakened or strengthened professional boundaries depending on their use.*	Participants utilised common biomedical terminology in their discussions to weaken boundaries. There were occasions where profession-specific jargon strengthened boundaries between COPs. Additionally, ambiguous terminology such as the concept of a ‘subluxation’ was discussed, which may have reinforced boundaries.
**3) Organisational boundary objects**	**3.1) Appointment fees and duration** *Differences with durations and fees for COP appointments reinforce boundaries.*	Chiropractic appointments were shorter in duration and had lower fees than those for physiotherapy or osteopathy. This strengthened the structural differences and boundaries between chiropractic and the others, while weakening the boundaries between physiotherapy and osteopathy.
	**3.2) Remuneration structure around joint consultations** *Clinic policies attempted to promote COP collaboration; however contractor arrangement may have reinforced boundaries.*	Policies were in place to promote and incentivise COP collaboration in both clinics. Contractor arrangements however may have de-incentivised COP collaboration, further reinforcing boundaries.
	**3.3) Health insurance benefit codes** Clinics had to, at times, treat the boundaries between COPs more fluidly to comply with health fund benefit requirements.	Health fund rebates are restricted to specific professions for specific services. One clinic had to use a more fluid definition of professional roles and boundaries in order to comply with these restrictions– enabling a chiropractor to run an exercise class which was normally run by a physiotherapist.

### Theme 1 - physical boundary objects within the clinic

At the broadest physical level, the first boundary object we observed was the floorplan and layout of each clinic into various arrangements of clinicians’ workspaces. In Clinic A participants had their own private rooms with doors lining both sides of a single long hallway through the centre of the clinic, interspersed by COP profession in no seemingly distinct order. From outside each door, there were no distinguishing physical features identifying them to one profession or another, thus potentially creating more fluid boundaries between the professions. Clinicians in Clinic A would go to the waiting room and bring patients to their rooms and close the door during a consultation, usually leaving their door open when not seeing a patient. By contrast, Clinic B layout was arranged differently, possibly reinforcing perceived professional boundaries:

*“The different ‘wings’ of the facility denote separation between different ‘departments’ and may serve to reinforce physical boundaries between the professions. While these areas are only meters away from each other, there seemed to be enough distance between them to have limited/minimal movement of clinicians from one area to another, limiting the incidental interactions they might have throughout a typical working day.”* (Clinic B field notes).

Within the designated areas or rooms in both clinics, there were different pieces of large equipment that were specific to each profession’s practice that served as boundary objects. They seemed to strengthen professional boundaries by essentially allowing only one profession to work in that space, rarely did they share rooms between professions. For example, in Clinic A the chiropractic rooms had ‘drop-tables’ as opposed to the physiotherapist’s rooms which had more rigid plinths - enough of a difference to restrict the use of each treatment table, and therefore room, to the specific profession. Demonstrating the influence of the ‘treatment table’ as a boundary object on the fluidity of professional boundaries, the osteopath in Clinic B utilised the same type of treatment table as the physiotherapists and his workspace was physically closer to the physiotherapists than the chiropractors. This example demonstrates a softening of the boundaries between the osteopath and the physiotherapists, while at the same time strengthening their boundary with the chiropractors. This physical boundary object was further reinforced by the osteopath’s beliefs about his work:

*“…he described his work as being more “structural” as opposed to “indirect or cranial” osteopathy. He stated that he works differently to everyone else in the clinic and offers a unique skillset, also saying that his work was different to the previous osteopath at the clinic who worked in the chiropractic wing and who (reportedly) treated very similarly to the chiropractors.*” (Clinic B field notes).

We can see here multiple layers of boundary-work where this participant, whether intentionally or not, is distinguishing himself both inter and intra-professionally, especially disassociating his work from that of the chiropractors.

Smaller pieces of equipment, used to apply some treatment intervention, were identified as boundary objects within the various workspaces. These physical objects, such as laser therapy machines, spring-loaded activators, and exercise bands, were used by participants at times to distinguish themselves even intra-professionally. They were usually located in specific areas of the clinic associated with each profession. The way participants discussed their use seemed to be connected to their beliefs about how others were different from themselves - an example of boundary-work. For example, some participant physiotherapists would question the way that another physiotherapist applied therapeutic exercise for his patients. Similarly, some of the chiropractic participants would describe how other chiropractors in the clinic were known to use some pieces of equipment whereas they didn’t, or vice versa. Below is an example of two contrasting statements from two different chiropractors about the same boundary object– the ‘activator’:

*“I asked one chiropractor if he uses the activator as a treatment modality, he said he used them on one person because they asked for it, but he doesn’t believe that it does much.”* (Clinic A field notes).

*“I asked a senior chiropractor about the activator, how it works. She went on to explain in depth for about 10-15mins the scientific evidence behind it, how it was discovered, and the positive effects it has had on many of her patients.”* (Clinic B field notes).

The contrasting views of two participants from the same profession about the same object may be explained by their age and experience - the first statement is from a younger chiropractor who did not believe the device “does much”, challenging its scientific efficacy and usefulness. Whereas the older and more experienced chiropractor felt strongly about its efficacy and usefulness in her own clinical experience. While more data is required to understand this further, it demonstrates the potential that differences in age and experience may be linked to differences in beliefs and standards with regards to evidence-based practice.

Taken together, these examples indicate that physical spaces and objects are not passively present in the clinics, instead they are active influencers on the nature of the boundaries between COPs. The relationships of COPs with these boundary objects constantly (re)create their social world, both strengthening and weakening boundaries between and within each profession.

### Theme 2 - social boundary objects: identities and discourse

A social boundary object that was observed was the concept of ‘professional identity’ and how it was discussed by participants. Depending on the presenting situation, each participant seemed to fluidly switch between two main identities: (1) clinician as a person with unique social and clinical skills; and (2) clinician as a registered member of a particular profession. These various layers of identity seemed to impact how they undertook boundary-work in different situations. Under normal circumstances, clinicians must outwardly identify themselves with a specific professional group when undertaking patient consultations and are licensed to work based on their registration with a particular profession (even in cases of dual registration). However, we observed in these settings that when a participant entered the social space of the clinic, individual identity (category 1 above) was a stronger determinant of how they were perceived by colleagues *over* professional registration (category 2). In other words, they believed and respected that each clinician has a unique personality, set of skills and preferences regarding how to practice. For example:

*“[physiotherapist] joined the discussion [with the chiropractor] and they both spoke about how they don’t focus too much on their professional titles… They seem to be moving away from the professional titles and more focused on their unique interests and skills.”* (Clinic A field notes).

*“The physiotherapist said that he refers to the chiropractor not necessarily because he is a chiro, but because he knows that he gets good results for people with headaches and enjoys that work, and that chiro refers to him for people with hamstring injuries because that is his specialty, as an example.”* (Clinic A field notes).

These excerpts demonstrate that the outward identity of professional title is being challenged by a more nuanced view of each clinician as a unique person. This softening of the boundaries around one’s professional registration demonstrates the fluidity of their identity in co-located situations, and therefore the fluidity of perceived professional boundaries in the clinic.

The next few examples demonstrate another social boundary object that we observed, namely the professional discourse. We are using the term ‘discourse’ here to encompass the knowledge and values that are conveyed through spoken language to create (shared) meaning. Within this context, we identified a professional discourse underpinned by biomedical aspects, which relied on medical and anatomical vocabulary and seemed to enhance communication and knowledge transfer between participants as they mostly understood each other’s intended meaning when discussing clients’ MSK conditions. Most participants used biomedical discourse when discussing their work with colleagues and clients, although they seemed to avoid profession specific jargon to enable clearer communication. This discourse appeared to take place within a comfortable environment, based on observed body language, tone of voice and eye contact, where participants could communicate judgement free, share similar beliefs about their work, which in turn may have boosted their confidence to collaborate. For example, during a joint consultation we observed:

*“After some muscle testing, the physio recommends some exercises to the patient, and they begin to practice them. While this is happening, the informal conversation between the patient and the two clinicians (from different professions) in the room is heavily anatomical, describing the specific structures causing pain, dysfunction, specific muscle names used, etc.”* (Clinic A field notes).

Although there was enough commonality within the overarching professional discourse to enable mutual understanding between COPs and potentially foster collaboration at times, specific jargons limited to a certain profession prevented COPs from initiating collaborative communication in the first place, resulting in stronger boundaries between them. For example:

*“I was near a chiropractor preparing to see a patient who had most recently been seen by the physiotherapist. He was quickly reviewing the progress notes and saw that the physio had written in the plan “review PRN”. He said under his breath that he didn’t know what PRN meant, so I told him that it means “as needed”… He then made a pointed comment that he would have just written “as needed.” I asked if he plans to check this unfamiliar term with the physio, and he said no and moved on with his work.”* (Clinic B field notes).

This example of a boundary object within written notes highlights a few key ideas about the nature of professional boundaries in the clinic. At one level, this example demonstrates the differences between the training between the physiotherapist and chiropractor. In Australia, physiotherapy students undertake clinical placements within hospitals, whereas chiropractors and osteopaths do not work in the public health system, offering one explicit distinction. At another level, it was interesting to observe how the clinic’s aspirations for interprofessional collaboration (see Sect. 3.1) were restricted by the practicalities of busy clinical practice– the chiropractor only had a few moments to quickly review what the physiotherapist had been doing with the patient who was now waiting to see them. There wasn’t enough time for him to clarify with the physiotherapist in person about the specific jargon, and there were likely other barriers such as distance and inconvenience. Additionally, the chiropractor held more power as he was older and more experienced than the physiotherapist, and he may have felt uncomfortable about approaching them to consult on a relatively trivial matter, challenging the power dynamic.

At times, the use of biomedical language created ambiguity and confusion, given the meanings that certain terms had for different professions. For instance, within the context of manual therapy of the spine, individual clinicians seemed to use certain terms differently. After asking a chiropractor about their understanding of the term ‘subluxation’, the researcher wrote:

“*He said that a previous chiropractor he worked for said the term subluxation will be phased out in the future. At university the use of the term was mixed– it was used to describe how/where you were adjusting someone, how you would take notes. He said that the medical use of the word is a partial dislocation, a chiropractic use of it describes where you are adjusting. This connects to the traditional philosophy where the nervous system is affected by subluxations– he added that this idea has been ‘debunked’.”* (Clinic A field notes).

In this interaction the chiropractor seems to highlight that there are different uses of the term ‘subluxation’ by different people and in different contexts, both within the chiropractic profession and between different professions. It is likely that the use of this term within the co-located clinic either verbally or within written notes may cause confusion, however this was not directly observed during the data collection. The boundary object of physical-therapy-related yet ambiguous terminology may simultaneously connect and separate the social worlds of the professions, demonstrating how professional discourses can make professional boundaries between COPs more fluid.

### Theme 3 - organisational boundary objects

There were several organisational factors that we considered as boundary objects that influenced the nature of professional boundaries in the clinic. One such factor related to appointment durations and fees for each profession. In both clinics, consultations were generally different between the professions, with physiotherapy and osteopathy subsequent appointments costing slightly more and lasting longer (20-30 min) than chiropractic appointments (usually 10-20 min). However, initial appointments for all three professions varied from 30 to 60 min. One director described several reasons for the different subsequent appointment times, including limitations imposed by public and private health funding organisations and the distinct nature and speed of treatment modalities associated with each profession. Additionally, the appointment fees for each profession were different. While the fees were lower for chiropractic appointments, the per hour rate was higher as compared to physiotherapy and osteopathy due to the shorter appointment times. This example demonstrates a strengthening of the differences, and therefore boundaries, between chiropractors and the others in these co-located environments, while weakening boundaries between physiotherapy and osteopathy in this case.

Another boundary object operating at the organisational level and influencing professional boundaries and collaboration was the clinic’s remuneration structure for each profession. The difference between how clinicians were paid for their work may have influenced how they performed boundary-work and the extent to which collaboration across boundaries was encouraged. Collaboration between participants occurred mostly through cross-referrals and occasionally in joint consultations. In both clinics, there were policies in place to encourage this kind of collaboration between the professions and discourage competition. For example, after a joint consultation, patients only pay one fee (based on the rate charged for the main provider) and both clinicians are paid by the clinic for that appointment. However, it was unclear how the salary structure, namely an hourly rate versus a commission-based pay, influenced the way clinicians perceived boundaries and collaboration. For example, being paid on commission for how many patients they might consult, may naturally de-incentivise the extent to which they spend time in joint consultations that earn them less and further reinforce professional boundaries:

*“One chiro was telling me that all the chiros are contractors– so the incentive to keep patients to yourself is much higher. He thinks that is a big factor in why they don’t spend much time together, he felt that it could be different. In the contractor arrangement, it makes sense to specialize and stake your claim on a particular area of work or ‘jurisdiction’, so others refer to you for that, e.g. sports, women’s health, headaches, etc.”* (Clinic B field notes).

One final example of an organisational boundary object influencing the nature of professional boundaries was the health insurance benefit codes and their impact on clinical practice. In Australia, health benefit codes established by government agencies and private health insurance companies limit the kinds of services that patients can claim financial rebates for when seeing COPs. Appointments with COPs are commonly pooled together into a fixed amount per year despite there being differences in the types of services each can provide. For example, patients attending group exercise classes that are run by physiotherapists can claim a health fund benefit, however, they cannot if it is being run by chiropractors or osteopaths. During our observations of one of the clinics, we observed that the clinic administration somewhat overrode professional boundaries, in this case allowing both the physiotherapist and chiropractor to alternate running the class, justifying it by ensuring the physiotherapist supervises the program. This example highlights the challenges of a clinic to practically comply with organisational boundary objects that reinforce boundaries between the professions, while simultaneously reducing their impact on the day-to-day operations of the clinic, weakening boundaries at that level. It demonstrates that while at a higher level the boundaries between professions are well defined in policy, at the clinic and clinician levels these boundaries become more fluid to facilitate increased economic benefit for the practice and accessibility for the patient.

We have considered here three organisational boundary objects and have seen in both clinics how they at times strengthen, and at other times weaken, professional boundaries between COPs as the participants navigate these imposed structures in their daily practice. These examples demonstrate the challenges of running a co-located clinic and the tensions that arise from trying to promote collaboration and fluidity, while at the same time adhering to regulatory constraints that attempt to reinforce professional boundaries.

## Discussion

This study aimed to shed light on the nature of professional boundaries between COPs in two co-located environments, and to explore and describe the ways in which these boundaries may be reinforced, weakened or navigated. We used the concept of boundary objects in our results to help us describe the nature of the boundaries between COPs. The results have illustrated that physical, social, and organisational boundary objects, both tangible and intangible, can make these boundaries fluid, at times shifting, strengthening, or weakening them. For example, physical boundary objects like the clinic’s layout and specific equipment both reinforced and weakened the perceived distinctions between each profession in the way the clinicians themselves spoke about their work. The dynamic and simultaneous reinforcement and weakening of boundaries highlighted their fluidity. Similarly, examples of social boundary objects, such as identity and professional discourse underpinned by biomedical aspects, demonstrated how clinicians perform boundary-work to fluidly switch between different situational identities and use comfortable biomedical language to identify more closely with, or distinguish themselves from, those from the other professions. The third group of boundary objects highlighted in the results were categorised as organisational, such as the clinic’s appointment and remuneration structure, and health insurance benefit codes. These have demonstrated how the ‘traditional’ professional boundaries between COPs influenced and were navigated by the clinic’s administration as they strived to adapt the policies to their reality and processes. Overall, our analysis suggests that in co-located clinical settings, the professional boundaries between COPs are more fluid than once thought.

In post-professional literature, there has been discussion suggesting the decentring of health professions, which challenges conventional concepts of professional power, identity and boundaries [29]. Post-professional theorising has now been documented in relation to physiotherapy [22] and osteopathy [24, 30], however it is yet to fully emerge within chiropractic. Our results showed that the way COPs in co-located settings identify themselves and each other in different ways, sometimes more strongly identifying with their profession and at other times more as a unique person with distinct beliefs, knowledge and skills. This may be due to their desire to ensure that their knowledge and skills remain relevant in different situations, or due to a weaker connection between their professional training/title and how they wish to practice or be seen as a practitioner. While our research does not directly investigate professional identity of COPs as its central aim, other research has investigated personal and professional identity of these three professions, including the influences and fluidity of these identities [31–33]. However, we acknowledge the interconnections between professional identity and boundaries, and the results show that the fluidity of the professional boundaries challenged traditional boundaries between the professions and recognised that individual clinicians are capable of a range of different beliefs and practices. The shifting of professional boundaries and decentring of the professional title in one’s work may be indicative of a post-professional movement within the COP professions. Based on this, common negative professional stereotypes, and the narratives that one profession tells of the others could also evolve to reflect the wide spectrum of clinical practice, and that there may be no way of knowing or controlling how each clinician reflects their professional title in their work. Future research could explore the impact of the post-professional movement on the education and practice of COPs.

Our findings showed that in these co-located settings the forces of the market and competition between COPs seemed to influence the day-to-day work of the clinicians and the professional boundaries between them. From a neo-Weberian perspective, which views the professions as social actors competing for social and economic prestige [34], the results showed examples where boundaries were reinforced through boundary objects such as the appointment fees and remuneration structure. For example, we can assume that each business remains financially viable whilst balancing competitive service prices for consumers and paying their staff well. The experience shared by one chiropractor being paid as a contractor and only earning based on the number of clients he sees, may decrease his willingness and capacity to engage in professional collaboration, thus reducing connection with staff from other professions, and subsequently reinforcing the professional and personal boundaries between him and others. While it may be uncommon to see inter-professional collaboration in private MSK practice [35], these examples showed that even for clinics with strong intentions to breakdown inter-professional boundaries and promote collaboration, external influences of competition and the market ensured that certain policies and practices were kept in place to remain economically viable, reinforcing professional boundaries.

There were some examples of boundary-work that emerged in the results that were emblematic of the wider discourse around evidence-based practice (EBP) and may have been influenced by the age and experience of participants. One example was how the osteopath (a recent graduate) attempted to distance his own beliefs from those of other osteopaths and his chiropractor colleagues, reportedly aligning his scientific beliefs and practice more with the physiotherapists. This was further demonstrated by his choice to work in a room physically closer to the physiotherapists and away from the chiropractors. Another example was where several young clinicians explained that unlike other clinicians in the clinic, they do not use certain external therapeutic devices because they do not believe in the scientific evidence behind them. While not a focus of our ethnographic study, these examples showed that less experienced participants, regardless of profession, were more likely to express more contemporary EBP beliefs than older, more experienced clinicians. This observation is echoed in other literature that has investigated EBP beliefs within chiropractic, osteopathy, and physiotherapy [36–38]. It may also signify changes in the curricula or cultures of training programs for these professions in recent years, however this would need to be further investigated. Considering this relationship between clinicians and their beliefs about EBP, we may be starting to see the glimmerings of a movement of groups of clinicians enclosing themselves within boundaries that are different to those of traditional professional boundaries, in this case dividing clinicians into groups based on EBP beliefs. While it is unclear what might contribute to the shifting relationship between EBP beliefs and professional boundaries of COPs, further research could investigate this.

Our research has shown that the boundaries between COPs are fluid in co-located clinical settings. What consumers, clinicians and administrators might traditionally see as three distinct professions may not be so in practice. This calls for deeper analysis and study of the reality of chiropractic, osteopathic and physiotherapy in practice, which in turn may help answer existential questions about the future of these and other manual therapy and MSK related professions in the post-professional era. At the very least, the public discourse around MSK healthcare should evolve to be less divisive and more open to the possibilities and diversity of clinicians practicing under the banner of any of these three professions. There is room for growth and education within each profession to appreciate that the beliefs and practices of clinicians from other professions may not be dissimilar to their own, or at least as dissimilar as they may believe.

### Limitations and Methodological Considerations

There are several limitations and methodological considerations for interpreting the findings of this study. First, our sample included mostly physiotherapists and chiropractors, with only one osteopath being observed in one of the clinics. The results presented here could have been different if more osteopaths with a range of experiences had participated, with more relevance for future studies seeking to investigate boundaries between COPs. Second, the study was conducted in Australia, with unique socio-cultural norms, as well as systems and regulations around these professions. Therefore, the transferability of our results may be limited to countries that have social norms and health systems similar to Australia. Third, we only investigated two clinics where COPs are co-located, which may not be reflective of other multidisciplinary clinics, as well as monodisciplinary clinics which far outnumber those where COPs are co-located. Therefore, clinicians in these other settings may think, speak and act quite differently to those documented here. Fourth, the study generated data pertaining to predominantly clinician-clinician interactions rather than clinician-patient interactions, and as such the team was not able to analyse and discuss other boundary objects such as the patient’s body, especially in cases of shared treatment, as a major site of interaction. Lastly, the research team comprised of three physiotherapists, one osteopath and no chiropractors. While this is an obvious limitation in that the direct perspective of a chiropractor was missing from the analysis and final manuscript, the team collectively had prior experience working and researching with chiropractors. The results presented here could have been different if the observer was a chiropractor or an osteopath and/or if a chiropractor was included in the research team.

## Conclusion

This study aimed to understand the nature of professional boundaries between chiropractors, osteopaths and physiotherapists (COPs) in co-located environments, through ethnographic observations of two clinics in Australia. Our qualitative reflexive thematic analysis produced findings suggesting that within these environments, the professional boundaries between COPs are fluid in nature, sometimes strengthening and at other times weakening depending on different factors. These factors were described as boundary objects, such as physical objects used in the clinic, how participants spoke about their professional identity, or even organisational influences on the operation of the clinics. Overall, this study has shown that the boundaries between COPs in co-located environments can be fluid, and this may have implications for future research on professional boundaries between COPs broadly in more clinical and non-clinical settings.

### Electronic supplementary material

Below is the link to the electronic supplementary material.

Supplementary Material 1 - Observation recording template

## Data Availability

The datasets generated and/or analysed during the current study are not publicly available due to the nature of the qualitative ethnographic data being quite detailed and descriptive that by sharing it publicly would be breaching confidentiality. However, parts of the data may be made available from the corresponding author on reasonable request.
