# Supplementary material for: Fluid professional boundaries: ethnographic observations of co-located chiropractors, osteopaths and physiotherapists
Source: BMC Health Serv Res. 2024 Mar 15;24:344. doi: 10.1186/s12913-024-10738-1 (PMC10943826; doi:10.1186/s12913-024-10738-1)
Supplement: Supplementary file 1 — Supplementary Material 1 - Observation recording template [file 12913_2024_10738_MOESM1_ESM.docx]

# Supplementary materials

## 1. Observation occasion template

| **Observation Information** | **Notes** |
| --- | --- |
| Observation occasion code | (e.g. O1) |
| Start and finish time | (include duration) |
| Location | (e.g. hallway, gym, break room) |
| Participants involved | (use pseudonyms) |
| Other people nearby | Yes/No (if yes, who?)  Verbal consent gained: Yes/No |
| Researcher body position during observation |  |
| Detailed environment description during observation | (e.g. physical, social, emotional) |
| Conversation notes | (e.g. what was said by participants that relates to the research questions? Voice, tone, emotions?) |
| Non-verbal communication | (e.g. participant positioning relative to each other, body language, facial expressions, etc.) |
| Questions asked by the researcher and responses |  |
| Any other relevant details |  |
